# Fatty Acid Profile of Blood Plasma at Mating and Early Gestation in Rabbit

**DOI:** 10.3390/ani11113200

**Published:** 2021-11-09

**Authors:** Imane Hadjadj, Anna-Katharina Hankele, Eva Armero, María-José Argente, María de la Luz García

**Affiliations:** 1 Centro de Investigación e Innovación Agroalimentaria y Agroambiental (CIAGRO-UMH), Miguel Hernández University, Ctra. de Beniel km 3,2., 03312 Orihuela, Spain; imane.hadjadj@alu.umh.es (I.H.); mj.argente@umh.es (M.-J.A.); 2 ETH Zurich, Animal Physiology, Institute of Agricultural Sciences, Universitaetstr. 2, 8092 Zurich, Switzerland; anna-katharina.hankele@usys.ethz.ch; 3 Department of Agronomic Engineering, Technical University of Cartagena, Paseo Alfonso XIII 48, 30203 Cartagena, Spain; eva.armero@upct.es

**Keywords:** embryo, MUFA, ovulation, PUFA, SFA

## Abstract

**Simple Summary:**

Fatty acids can be used as an energy substrate by oocytes and embryos. Ovulation rate and normal preimplantation embryos are limiting factors to increased litter size in rabbits. Knowledge of the fatty acid profile in blood plasma at mating and in early gestation and its relationship with the ovulation rate and early embryonic development can help improve doe productivity. In our study, palmitic, linoleic, oleic and stearic acids show the highest concentrations. Moreover, monounsaturated fatty acids are correlated with ovulation rate and normal embryos. The more SFA, the greater the embryonic development. This study could be useful for designing enriched feeds in animal production and for embryological studies, as the rabbit is an experimental model.

**Abstract:**

The aim of this study was to analyse the fatty acid (FA) profile of blood plasma at mating and 72 hpm by gas chromatography. Moreover, the correlation between FA and ovulation rate, normal embryos and compacted morulae was estimated. Palmitic, linoleic, oleic and stearic were the highest FA concentrations at mating and 72 hpm. Most long chain saturated and PUFA were higher at 72 hpm than at mating, while MUFA were higher at mating. SFA, MUFA and PUFA were high and positively correlated. Correlation was 0.643 between MUFA at mating and ovulation rate, and 0.781 between MUFA and normal embryos, respectively. Compacted morulae were slightly correlated with SFA at mating (0.465). In conclusion, the FA profile of plasma varies depending on the reproductive cycle of the rabbit female, adapting to energetic requirements at mating and early gestation. Moreover, positive correlations are found between fatty acids and ovulation rate and embryo development and quality.

## 1. Introduction

From ovulation to implantation, oocytes and embryos are supported by maternal secretions from the oviduct and uterus (cow [1], sheep [2], murine [3], horse [4], sow [5] and rabbit [6]). The oviductal and uterine fluids mainly originate from blood filtrates [7] and secretions of endometrial luminal and glandular epithelial cells [8]. Thus, a complex milieu containing proteins, amino acids, carbohydrates and fatty acids (FA) is constituted to maintain embryo viability.

Fatty acids represent compact reserves of stored energy for oocytes and embryos [9]. Additionally, FA play a crucial role in modifying the physical properties and functions of biological membranes, and have potential effects on oocyte growth and maturation and embryo development and transport through reproductive traits [10,11,12]. The ability for the exogenous uptake of FA by the embryo has been demonstrated in several studies [13,14,15]. Specifically, rabbit embryos can already absorb FA at zygote stage [13], and it is reported that exogenously supplied fatty acids are beneficial for growth and development in cultivated oocytes and embryos [10]. Moreover, FA enriched diets consumed around the fertilization period and the first days of pregnancy and conditions in the oviductal and uterine environment [14] can affect embryo quality [15]. Rabbit is a livestock species reared for the production of hair, skin or meat and also as an experimental reference for other species, such as pigs or humans [16]. A detailed understanding of how ovulation rate and early embryo survival and development are affected by the FA profile could improve the productivity of rabbit females and further basic knowledge as an experimental model. Therefore, the aim of this work was to study the fatty acid profile at mating and in early gestation and its relationship with ovulation rate and embryo quality and development in rabbits.

## 2. Materials and Methods

### 2.1. Experiment Animals and Design

A total of 15 non-lactating multiparous rabbit females were used. Does were 9–10 months of age. Females belonged to two lines selected divergently by litter size variability [17]. Females were kept on the experimental farm at the Universidad Miguel Hernández de Elche (Spain). They were reared in individual cages and fed ad libitum with a commercial diet (crude protein, 17.5%; crude fibre, 15.5%; ether extract, 5.4%; ash, 8.1%) during their reproductive life.

### 2.2. Blood Sampling

Females that had finished their fourth lactation were weighed and mated. Following the blood sampling procedure described in [18], two blood samples of 3 mL were drawn from the central artery of each doe’s ear at mating and 72 h post-mating (hpm). The blood sample was collected into a tube with tripotassium ethylenediaminetetraacetic acid (K3-EDTA). All samples were immediately centrifuged at 4000× *g* rpm for 15 min, and plasma was stored at −80 °C until required for lipid analyses.

### 2.3. Reproductive Traits

At 72 hpm, females were euthanized by intravenous administration of sodium thiopental in a dose of 50 mg/kg of body weight (Thiobarbital, B. Braun Medical S.A., Barcelona, Spain). The entire reproductive tract was immediately removed in order to measure reproductive traits.

Total ovulation rate (OR) was estimated as the number of corpora lutea. Total embryos were recovered by perfusion of oviducts and uterine horns with 10 mL of Dulbecco’s phosphate-buffered saline containing 0.2% of BSA. Embryos were classified as normal embryos (NE) when they presented homogeneous cellular mass and intact embryo coat, using a binocular stereoscopy microscope (Leica Mz 9.5; ×600). Embryos were classified as early morulae or compacted morulae. Compacted morulae (CM) were expressed as percentage of NE.

### 2.4. Fatty Acid Analyses

All samples were analysed in duplicate. A 200 µL sample of plasma was taken in a screw cap glass tube. One milliliter of 0.5 M NaOH-methanol was added to each sample and the sample was boiled at 90 °C for 15 min. The sample was cooled in an ice bath to room temperature, 2 mL of BF3-methanol was added, and the sample was boiled at 90 °C for 20 s. The sample was again cooled in an ice bath to room temperature, 1 mL of isooctane was added, and the mixture was shaken. In the measurement of recovery ratio of the internal standard, 1 mL iso-octane containing 0.05 mg 13:0 ME and 0.01 mg 19:0 ME was added. Five milliliters of saturated NaCl solution was then added, and the mixture was centrifuged at 4000× *g* rpm for 10 min at 4 °C. When the iso-octane layer separated from the aqueous lower phase, the iso-octane layer was transferred to a glass vial. After iso-octane was evaporated under a stream of nitrogen gas, 250 µL of hexane was added, and the sample was injected into the GC system. The fatty acids were measured using a gas chromatograph (GC-17A, Shimadzu, Kyoto, Japan) coupled with a flame ionization detector (FID) equipped with a capillary column (CP Sil 88 100 m × 0.25 mm i.d., 0.20 µm film thickness; Agilent technologies, Madrid, Spain). The carrier gas was helium (flow 1.2 mL/min) with a split injection of 1:1. The temperature profiles were as follows: initial temperature, 45 °C for 4 min; heating first rate, 13 °C/min until 175 °C (27 min isolation) and a second rate, 4 °C until 215 °C (30 min isolation); injector temperature, 250 °C; and detector temperature, 260 °C. The fatty acids were identified by comparing the retention times with those of Fame Standard Mix (CRM47885, Sigma Aldrich, St. Louis, MA, USA).

### 2.5. Statistical Analyses

The model for analyzing FA profile included the effects of moment (two levels: at mating and at 72 hpm), line, random effect of female and weight of female as covariate.

All analyses were performed using Bayesian methodology [19]. Bounded uniform priors were used for all effects with the exception of the female effect, which was considered normally distributed, with mean 0 and variance I$\sigma_{f}^{2}$, where I is a unity matrix, and $\sigma_{f}^{2}$ is the variance of the female effect. Female and residual effects were considered to be independent. Residuals were a priori normally distributed, with mean 0 and variance I$\sigma_{e}^{2}$. The priors for the variances were also bounded uniform. Features of the marginal posterior distributions for all unknowns were estimated using Gibbs sampling. The Rabbit program developed by the Institute for Animal Science and Technology (Valencia, Spain) was used for all procedures. We used a chain of 60,000 samples, with a burn-in period of 10,000. Only one out of every 10 samples was saved for inferences. Convergence was tested using Geweke’s Z criterion and Monte Carlo sampling errors were computed using time-series procedures.

Residual correlations between FA at mating and at 72 hpm after correction by line and weight of female were estimated. A principal component analysis was carried out. These analyses were performed using the SAS statistical package.

## 3. Results

Table 1 shows the features of the marginal posterior distributions of the difference between FA profile measured at mating and at 72 hpm. A total of 31 different FA were identified in blood plasma, and three FA were not detected (octanoic, cis-10 pentadecanoic and adrenic acid). The highest FA concentrations at mating and at 72 hpm were palmitic (425.01 ng/mL), linoleic (408.00 ng/mL), cis-9 oleic (385.30 ng/mL) and stearic (238.08 ng/mL), while palmitoleic (29.31 ng/mL), heptadecanoic (21.26 ng/mL), α-linolenic (13.77 ng/mL), trans-9 elaidic (13.41 ng/mL), myristic (12.91 ng/mL) and pentadecanoic (12.28 ng/mL) were in lower concentration.

Short and medium chain FA were shown in very low concentrations and similar concentrations were found at mating and at 72 hpm. Only lauric was higher at mating than at 72 hpm (D = + 0.39 ng/mL; *p* > 0.80).

Most long chain fatty acids were higher at 72 hpm than at mating. Myristic, pentadecanoic, arachidic, heneicosanoic, behenic and tricosylic increased their concentration in this period. Conversely, heptadecanoic and lignoceric were higher at mating than at 72 hpm. Similar concentrations were found for palmitic, stearic and SFA between both moments.

Monounsaturated FA (MUFA) were higher at mating than 72 hpm (D = + 55.75 ng/mL; *p* > 0.80). The increases ranged from 8% to 54% for myristoleic, trans-9 elaidic, cis-9 oleic, palmitoleic and cis-10 heptadecanoic.

Polyunsaturated FA (PUFA) showed a relevant lower concentration at mating than at 72 hpm (D = −30.67 ng/mL, *p* > 0.90). Specifically, these PUFA were linolelaidic (D = −1.26 ng/mL), linoleaic (D = −21.70 ng/mL), α-Linolenic (D = −4.61 ng/mL), cis−11,14 eicosadienoic (D = −1.41 ng/mL) and arachidonic (D = −1.09 ng/mL). Only γ-Linolenic was higher at mating, while similar concentrations were found for cis-11,14,17 eicosatrienoic and cis-4,7,10,13,16,19 docohexaenoic. 

**Figure 1:**
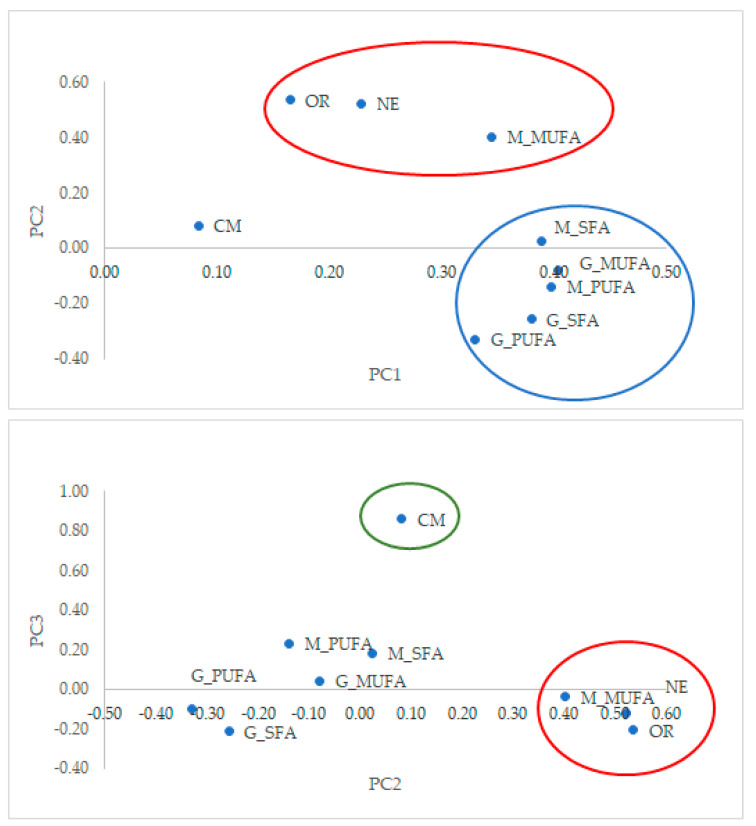
Projection of the traits in the plane defined by the 1st and 2nd principal component (PC) and the 2nd and 3rd PC. Variables of the 1st, 2nd and 3rd PC are surrounded by a blue, red and green circle, respectively. M_SFA: saturated fatty acid (FA) at mating; M_MUFA: monounsaturated FA at mating; M_PUFA: polyunsaturated FA at mating; G_SFA: saturated FA at 72 hpm; G_MUFA: monounsaturated FA at 72 hpm; G_PUFA: polyunsaturated FA at 72 hpm; OR: ovulation rate; NE: normal embryos; CM: compacted morulae.

The correlations between FA at mating and at 72 hpm and reproductive traits are presented in Table 2. Moreover, a principal component analysis was performed (Figure 1). The first three principal components explained 83% of total variation (53%, 18% and 12%, respectively). The predominant variables defining the first principal component were SFA, MUFA and PUFA both at mating and at 72 hpm, except for MUFA measured at mating. They were far from the origin and close to the axis. Thus, high and positive correlations were found between them, ranging from 0.492 to 0.899. These correlations were significant, except for MUFA at mating and PUFA at 72 hpm (Table 2).

Ovulation rate, normal embryos and MUFA at mating were located near the second principal component and close to each other (Figure 1). Thus, correlation between ovulation rate and normal embryos was 0.630. Correlation was 0.643 between MUFA at mating and ovulation rate, and 0.781 between MUFA and normal embryos, respectively (*p* < 0.05).

Compacted morulae are the predominant variable defining the third principal component. Compacted morulae were slightly correlated with SFA at mating (0.465), and uncorrelated with the other FA.

## 4. Discussion

The results of this study provide a detailed FA profile at mating, when ovulation takes place and in early gestation. Palmitic, linoleic, oleic and stearic acid are the highest FA concentrations at mating and 72 hpm. It has been reported that oleic, palmitic and linoleic are capable of supporting growth of one-cell rabbit embryos to viable morulae [10], as these FA may serve as a storage pool of metabolic precursors presented in oviductal and uterine fluids and embryos [20].

As expected, arachidonic acid was found in lower concentration at 72 hpm. Arachidonic acid is a crucial precursor of prostaglandins [8], which are found in low concentration in the first days of pregnancy due to their luteolytic action [21,22].

The absence or low concentration of short and medium FA, docohexaenoic and adrenic acid agrees with the findings of other studies [23].

The FA profile is different at mating than at 72 hpm. MUFA concentration is higher at mating, while PUFA is in higher concentration at 72 hpm. MUFA are mainly used by follicle components as primary energy sources (see review in [24]), whereas PUFA are nutritionally essential for embryo development [20]. High quality oocytes exhibit high levels of oleic acid (in cows, [25]). The PUFA concentrations, especially linoleic acid, are high not only in plasma but also in oviductal fluid and embryos [20]. PUFA in general, and linolenic acid in particular, support essential development processes in mammalian embryos [26]. Linoleic acid acts as a precursor for eicosanoids and regulates the processes of endocytosis or exocytosis, ion channel modulation, DNA polymerase inhibition and gene expression [27]. Moreover, linoleic acid stimulates protein kinase C, which is necessary for cell differentiation and growth [28,29]. In mammals, the absence of enzymes to introduce double bonds at carbon atoms beyond C-9 in FA chains determines linoleic acid as essential FA because they are not able to synthesize it. Thus, it must be included in the diet [30]. In this sense, rabbit females can increase deposition of PUFA in the periovarian adipose tissue after a long-term dietary supplementation with fish oil. This deposition could favour the PUFA accessibility to their ovarian structures as corpora lutea, whose activity, measured by the progesterone production, is increased during embryo preimplantation period [31].

To the best of our knowledge, this is the first time that the correlation between FA profile at mating and 72 hpm and ovulation rate and normal embryo and development have been studied. Ovulation rate and number of normal embryos are positively correlated with MUFA at mating. Moreover, the correlation between the percentage of compact morulae and SFA is slightly positive. Overlap of different embryo developmental stages is commonly observed [32] and could be related to the duration of ovulation or the oviductal and uterine fluid compositions [33]. Early morulae and compacted morulae can be found at 72 hpm. Palmitic acid, as the main SFA, was identified in abundant concentrations in more developed embryos [27] and is reported to be essential for FA elongation and desaturation in embryo development [34]. Moreover, incubation with palmitic acid showed that embryos can oxidize palmitic acid even at the single-cell stage, with subsequent increases, particularly from four-cell embryos onwards [20].

The results obtained in fatty acid composition could add useful knowledge of ovulation and early embryo metabolism. These data may also be useful practically in helping to develop non-destructive tests of oocyte and embryo quality and in improving culture media, cryopreservation and the success of IVF treatment.

## 5. Conclusions

The fatty acid profile of plasma varies depending on the reproductive cycle of the rabbit female, adapting to energy requirements at mating and in early gestation. In addition, correlations are found between fatty acids and ovulation rate and embryonic development and quality.

## Figures and Tables

**Table 1:** Means of the estimated marginal posterior distribution of the differences between mating and at 72 hpm for fatty acid profile (D).

Pathway	Biochemical Name		
		Means (ng/mL)	D (ng/mL)
Short chain fatty acids	Butyric (C4:0)	0.42	+0.08
	Hexanoic (C6:0)	0.02	+0.00
Medium chain fatty acids	Octanoic (C8:0)	ND	
	Decanoic (C10:0)	0.30	+0.06
	Undecanoic (C11:0)	0.45	+0.09
	Lauric (C12:0)	1.84	+0.39
Long chain fatty acids	Myristic (C14:0)	12.91	−5.57
	Pentadecanoic (C15:0)	12.28	−1.04
	Palmitic (C16:0)	425.01	+4.80
	Heptadecanoic (C17:0)	21.26	+2.14
	Stearic (C18:0)	238.08	−5.19
	Arachidic (C20:0)	3.50	−0.76
	Heneicosanoic (C21:0)	2.12	−0.98
	Behenic (C22:0)	5.08	−2.05
	Tricosylic (C23:0)	8.09	−5.55
	Lignoceric (C24:0)	0.85	+0.61
	ΣSFA	733.96	−14.61
Monounsaturated fatty acids	Myristoleic (C14:1c9)	2.63	+0.80
	Cis-10 pentadecenoic (C15:1c10)	ND	
	Trans-9 elaidic (C18:1t9)	13.41	+5.75
	Cis-9 oleic (C18:1c9)	385.30	+32.13
	Palmitoleic (C16:1c9)	29.31	+8.75
	Cis-10 heptadecenoic (C17:1c10)	5.91	+2.22
	Cis-11 eicosenoic (C20:1c11)	4.04	+0.41
	Erucic (C22:1c13)	0.25	+0.07
	Nervonic (C24:1c15)	2.17	+0.42
	ΣMUFA	445.07	+55.75
Polyunsaturated fatty acids	Linolelaidic (C18:2t9t12)	3.14	−1.26
	Linoleic (C18:2c9c12)	408.00	−21.70
	γ-Linolenic (C18:3c6c9c12)	2.23	+0.81
	α-Linolenic (C18:3c9c12c15)	13.77	−4.61
	Cis-11,14 eicosadienoic (C20:2)	6.65	−1.41
	Cis-11,14,17 eicosatrienoic (C20:3)	4.14	+0.84
	Arachidonic (C20:4c5c8c11c14)	1.61	−1.09
	Cis-4,7,10,13,16,19 docosahexaenoic (C22:6c4c7c10c13c16c19)	0.77	+0.02
	Adrenic (C22:4c7c10c13c16)	ND	
	ΣPUFA	440.76	−30.67

ND: not detected; SFA: saturated fatty acids; MUFA: monounsaturated fatty acids; PUFA: polyunsaturated fatty acids; Red: probability of the difference between mating and 72 hpm being > 0 when D > 0 or being < 0 when D < 0 is higher than 0.9; Green: probability of the difference between mating and 72 hpm being > 0 when D > 0 or being < 0 when D < 0 ranges from 0.80 to 0.90.

**Table 2:** Correlations for saturated fatty acid (SFA), monounsaturated fatty acid (MUFA), polyunsaturated fatty acid measured at mating and at 72 h post-mating, ovulation rate (OR), normal embryos (NE) and compacted morulae (CM).

		Mating		72 hpc					
		MUFA	PUFA	SFA	MUFA	PUFA	OR	NE	MC
Mating	SFA	0.675 *	0.899 *	0.813 *	0.808 *	0.543 *	0.267	0.268	0.465 ^+^
	MUFA		0.605 *	0.492 ^+^	0.721 *	0.342	0.643 *	0.781 *	0.169
	PUFA			0.807 *	0.861 *	0.740 *	0.123	0.341	0.328
72 hpc	SFA				0.826 *	0.773 *	0.092	0.267	−0.123
	MUFA					0.727 *	0.275	0.334	0.183
	PUFA						0.108	0.092	0.050
	OR							0.630 *	0.027
	NE								0.047

* Significant at level 0.05; ^+^ significant at level 0.10.

## Data Availability

Data are available upon request to the corresponding author.
